# Levels of omega-3 fatty acid in serum phospholipids and depression in patients with lung cancer

**DOI:** 10.1038/sj.bjc.6602877

**Published:** 2005-11-22

**Authors:** M Kobayakawa, S Yamawaki, K Hamazaki, T Akechi, M Inagaki, Y Uchitomi

**Affiliations:** 1 Psycho-Oncology Division, National Cancer Center Research Institute East, 6-5-1 Kashiwanoha, Kashiwa-City, Chiba 277-8577, Japan; 2 Psychiatry Division, Graduate School of Biomedical Science, Hiroshima University, 1-2-3 Kasumi, Minami-Ku, Hiroshima-City, Hiroshima 734-8551, Japan; 3 Division of Clinical Application, Department of Clinical Sciences, Institute of Natural Medicine, Toyama Medical and Pharmaceutical University, 2630 Sugitani, Toyama-City, Toyama 930-0194, Japan; 4 Department of Psychiatry, Nagoya City University Medical School, 1 Kawasumi, Mizuho-Cho, Mizuho-Ku, Nagoya-City, Aichi 467-8601, Japan; 5 Psychiatry Division, National Cancer Center Hospital East, 6-5-1 Kashiwanoha, Kashiwa-City, Chiba 277-8577, Japan

**Keywords:** omega-3 fatty acid, serum phospholipids, major depression, minor depression, lung cancer

## Abstract

Previous studies suggested that omega-3 fatty acids (FAs) have therapeutic effects against depression, but there is no evidence in the oncological setting. Our preliminary study reported the association between lower omega-3 FA intake and occurrence of depression in lung cancer patients. To explore the association further, the present study examined whether depression was associated with lower levels of omega-3 FAs in serum phospholipids. A total of 717 subjects in the Lung Cancer Database Project were divided into three groups by two cutoff points of the Hospital Anxiety and Depression Scale depression subscale (HADS-D). In all, 81 subjects of the nondepression and minor depression groups (HADS-D<5 and 5⩽HADS-D⩽10, respectively) were selected to match with 81 subjects of the major depression group (HADS-D>10) for age, gender, clinical stage, and performance status. Fatty acids were assayed by gas chromatography and compared among the three matched groups. There were no differences between the major depression group and nondepression group in any FAs. The minor depression group had higher mean levels of docosahexaenoic acid (mean±s.d. (%), nondepression: 7.40±1.54; minor depression: 7.90±1.40; major depression: 7.25±1.52, *P*=0.017). These results suggested that serum FAs are associated with minor, but not major, depression in lung cancer patients.

Major and minor depressions are one of the most common psychiatric problems in clinical oncological settings (Derogatis *et al*, 1983; Razavi and Stiefel, 1994; McDaniel *et al*, 1995). Docosahexaenoic acid (DHA), one of the omega-3 fatty acids (FAs), is a major component of membrane FAs in brain neural cells and lack of DHA has been shown to alter neural transmission in animal studies (Bruinsma and Taren, 2000; Innis, 2000; Ng and Innis, 2003). Previous studies have shown associations between DHA or its precursor, eicosapentaenoic acid (EPA, omega-3 FA), and major depression in subjects without cancer (Edwards *et al*, 1998; Hibbeln, 1998; Peet *et al*, 1998; Maes *et al*, 1999). Other studies have shown their possible therapeutic potential with few adverse effects in clinical trials (Nemets *et al*, 2002; Peet and Horrobin, 2002; Su *et al*, 2003). In the oncological setting, the use of antidepressants has been limited due to their adverse effects (Akizuki *et al*, 2002; Fisch, 2004). In our previous pilot study to obtain information for planning interventional research, depression in cancer patients was not associated with either EPA or DHA intakes, but was associated with lower intake of alpha-linolenic acid (ALA: major omega-3 FAs in diet, precursor of EPA and DHA) (Suzuki *et al*, 2004). To elucidate any such association in detail, the present study was designed to examine our hypothesis that lower levels of omega-3 FAs (EPA, DHA) in serum phospholipids are associated with major and minor depression in lung cancer patients.

## MATERIALS AND METHODS

### Study design and subjects

The present study was a three matched group case–control study, based in part on the Lung Cancer Database Project, which was a prospective cohort study for investigation of the pathogenesis of and development of new therapy for lung cancer at the National Cancer Center Hospital East and the National Cancer Center Research Institute East, Japan (Suzuki *et al*, 2004). The project, including the present study, was approved by the Institutional Review Board and the Ethics Committee of the National Cancer Center, Japan. All patients provided their written informed consent prior to enrolment in this study. The inclusion criteria of the present study, containing those of the Lung Cancer Database Project, were as follows: (1) patients who had visited the National Cancer Center Hospital East for their clinically diagnosed but never treated primary lung cancer between July 1999 and November 2001; (2) patients who had been informed of their lung cancer diagnosis; (3) patients who were physically able to complete questionnaires; and (4) patients who provided the blood samples. The exclusion criteria of the study were: (1) patients who had cognitive impairment, such as dementia and delirium; (2) patients whose medical attendants decided that their participation in the study had problems in clinical service; (3) patients whose clinical diagnosis of lung cancer had changed after a pathological report; (4) patients who had a performance status of more than 2 points (Eastern Cooperative Oncology Group) assessed by oncologists (to avoid affecting the results due to severe physical condition); (5) patients who had any past or current psychiatric disorder, except for depression, according to their medical chart; (6) patients who were taking antidepressants, major tranquilisers, and drugs for thyroid disease according to their medical chart (to exclude the effect on results by way of modifying depression); (7) patients who had brain neoplasms or brain metastasis diagnosed by head CT scan or MRI tests; and (8) patients who had a cerebral infarction or hemorrhage diagnosed from the examination of CT scan or MRI, or by clinical symptoms. During the corresponding period, 1013 patients were admitted to the Thoracic Oncology ward, National Cancer Center Hospital East, Japan. In all, 42 patients were ineligible, because of their poor physical conditions. Also, 30 patients refused to participate in this study and 39 patients could not be contacted (e.g., because of emergency admission and the immediate start of treatment). Of the remaining 902 patients, 829 patients provided blood samples and self-reported questionnaires, including the Hospital Anxiety and Depression Scale (Zigmond and Snaith, 1983). Based on all the exclusion criteria, 717 patients were eligible for enrolment in the present study.

### Measurement of depression

The self-reported questionnaires including the HADS were completed during the waiting period prior to admission, and were collected after admission. We used the HADS, which consists of seven-item anxiety and seven-item depression subscales, to assess anxiety and depressive symptoms during the preceding week in medically ill patients. The Japanese version of HADS was validated as having a good correlation of scores between test and retest (*r*=0.82) with Japanese subjects at the National Cancer Center Hospital East, Japan (Kugaya *et al*, 1998). In this study, depression was evaluated using the Hospital Anxiety and Depression Scale (HADS-D), with two cutoff points of 10 out of 11 and four out of five. These cutoff points have been found to yield a good sensitivity and specificity for the screening of depression (10 out of 11; 82.4 and 95.1%, major depression only, four out of five; 91.5 and 58.0%, adjustment disorder and major depression, respectively) (Kugaya *et al*, 1998). In the present study, subjects with higher scores on the HADS-D (>10) were assigned to the major depression group, those with middle scores on the HADS-D (5–10) formed the minor depression group, and those with lower scores on the HADS-D (<5) were placed in the nondepression group. By this classification, all eligible samples (*n*=717) were divided into three groups: the major depression group (*n*=81), the minor depression group (*n*=319), and the nondepression group (*n*=317).

To select cases and controls for comparisons among the three groups, all subjects in the major depression group (*n*=81), which was the smallest group of the three, were selected as standards to collect the same numbers of matched subjects in the minor depression and nondepression groups matched by gender, age, performance status (PS: 0 or 1), and the clinical stage assessed by TNM classification (Ia-IIIa or IIIb-IV). These subjects were pair-matched by gender and age (within 5 years). PS and clinical stage were considered as confounders on cancer patients, and were exactly matched among the three groups. As the major depression group included relatively more subjects at an advanced clinical stage, selected subjects in the other two groups (*n*=162) matched with those in the major depression group had a worse PS (*P*<0.001) and felt breathlessness more frequently (*P*=0.028) than the unselected subjects in the minor and nondepression groups (*n*=474), but apart from that there were no significant differences in any other factors including gender, age, and quantity of FA intake assessed by the Food Frequency Questionnaire (FFQ) (Tsubono *et al*, 1996) between the selected and the unselected subjects (data not shown).

### Measurement of FA composition of serum phospholipids

Following an overnight fast, blood samples for this study were collected early in the morning, a few days after admission, from all participating subjects by registered nurses, together with those for clinical examinations. Serum FAs were reflected by dietary FA intake in the past 3 weeks (Zock *et al*, 1997). After storing the samples for about 2 h at 4°C, serum was separated by centrifugation (1870***g***, 10 min), and stored at −80°C till measurements (4–5 years). Although, the present study did not confirm the influence of long storage up to 5 years, a previous study indicates good reproducibility to assay FA composition in serum samples stored up to 12 years at −80°C (Zeleniuch-Jacquotte *et al*, 2000). The FA composition of the total phospholipids fraction in serum was determined by experts (ST and SF) using gas chromatography. Briefly, FAs were extracted by the method of Bligh and Dyer (1959); the total phospholipids fraction was separated by thin-layer chromatography; after transmethylation with HCl-methanol, the FA composition was analysed by gas chromatography (GC14A Shimadzu Corporation, Kyoto) with a capillary column DB-225 (inside diameter 0.25 mm, 30 m in length, 0.25 *μ*m film thickness; J&W Scientific, Folsom, CA, USA); column temperature was kept at 170°C for the first 1 min, raised to 220°C at the rate of 4°C min^−1^, and kept at this temperature for 22 min; and the whole system was controlled with gas chromatography software, CLASS-GC10 ver. 1.3 (Shimadzu Corporation, Kyoto, Japan). The intra-assay coefficients of variance for EPA and DHA were 5.1 and 3.4%, respectively (*n*=10).

### Assessment of demographical and medical backgrounds

Information about clinical, demographic and social factors was collected from the database and medical chart in the same way as in our previous study (Suzuki *et al*, 2004). These data contained the following: gender, age, clinical staging assessed by TNM classification, performance status, educational level (longer than 9 years), smoking status (current smoker, ex-smoker, or nonsmoker), alcohol consumption estimated by FFQ, degree of breathlessness and pain (present or absence), and body mass index.

### Statistical analysis

For assessment of background factors, differences of continuous or categorical variables were analyzed by one-way analysis of variance (ANOVA) or the *χ*^2^-test, respectively.

The FA compositions of serum phospholipids were analysed by one-way ANOVA and analysis of covariance (ANCOVA). When any significant differences existed among the three groups, the Tukey HSD *post-hoc* analyses were performed. The background variables that were different in three groups were examined for any relationship with FA composition by the Student’s *t*-test (for categorical variables) or the Spearman’s rank correlation coefficient (for continuous variables). Only factors that were related to both background and FA compositions were set as covariates for the ANCOVA. All tests were two-tailed, with a *P*-value of <0.05 as the statistically significant level. Statistical analyses were performed using the statistical software package SPSS for Windows (Version 11.0J, SPSS Japan Institute Inc.). As no study exists on serum FAs in cancer patients with depression, the sample size was estimated by referring to previous studies of depressive noncancer patients (Maes *et al*, 1999; Frasure-Smith *et al*, 2004). According to the previous studies, the differences of the mean values of EPA and DHA between subjects with depression and normal controls were 22 and 14%, respectively. These differences were equivalent to the effect size of 0.45 by using the data of means and standard errors on FA compositions in Japanese healthy males (Kobayashi *et al*, 2001). The final size of samples needed for this study was calculated at 77 in each group with setting of the effect size at 0.45, with a two-tailed level of *α* at 0.05, and value of *β* at 0.20.

## RESULTS

### Backgrounds of matched subjects

Table 1 shows the background variables, gender, age, clinical stage, and performance status, which were used for matching, and also contains the educational level, smoking status, alcohol consumption, and body mass index. None of them showed significant differences among the three groups. Pathology of lung cancer (*χ*^2^=7.32, *P*=0.50), presence of breathlessness (*χ*^2^=5.87, *P*=0.058), and presence of pain (*χ*^2^=0.20, *P*=0.90) were also concerned as variables; however, none of them showed significant difference. The mean, median, standardised deviation, and range of duration between the time points of performing the HADS and blood sampling of all subjects were 3.6, 2.0, 5.0, and 0–33 days, respectively. The durations were not significantly different among the three groups (*F*=0.54, *P*=0.58).

### Fatty acid compositions among the three groups

Table 2 shows the results of the ANOVA and the mean values for the compositions of each of the omega-3 FAs in the three groups. Significant differences were shown for DHA (*F*=4.15, *P*=0.017), but no difference was seen in EPA levels (*F*=1.16, *P*=0.31) among the three groups. *Post-hoc* analyses, contrary to our hypothesis, failed to show any statistically significant difference between the major and nondepression groups, but the minor depression group had significantly higher levels of DHA composition (mean, s.d. 7.90%, 1.40) than the major depression group (7.25%, 1.52, *P*=0.017). The levels of DHA in the minor depression group were higher than those in the nondepression group, although the *P*-value did not show a statistical significance (7.40%, 1.54, *P*=0.09). There were also significant differences in docosapentaenoic acid (22:5n-3) (*F*=5.29, *P*=0.006) and total omega-3 FAs (*F*=3.91, *P*=0.021). There were no significant differences in ALA (data not shown) and ratios of total omega-6 FAs to total omega-3 FAs (data not shown).

As no variables could be identified with significant difference in the three groups, no factor was detected as a covariate. In addition to the main analyses, stratified analyses by gender were also performed, but these results were not so different from those of the main analyses (data not shown).

## DISCUSSION

As far as we know, this is the first study investigating an association between the levels of FAs in serum phospholipids and depression in patients after diagnosis of their lung cancer.

There was no significant difference in the levels of EPA and DHA in serum phospholipids between the major and nondepression groups, whereas the level of DHA was significantly higher in the minor depression group. Our hypothesis that these FAs would be lower in subjects with major and minor depression was therefore not supported.

In our previous cross-sectional study to investigate the relation between depression and omega-3 FA intake in patients with lung cancer, no significant differences in EPA or DHA intake were observed between subjects with depression (HADS-D⩾5) and subjects without depression (HADS-D⩽4). However, subjects with depression had significantly lower intake of ALA, which was a major dietary omega-3 FA (Suzuki *et al*, 2004). As ingested ALA can be converted into EPA and DHA *in vivo*, the result of our previous study (Suzuki *et al*, 2004) did not seem to be inconsistent with the studies indicating lower levels of EPA and DHA in phospholipids in depression (Edwards *et al*, 1998; Peet *et al*, 1998; Maes *et al*, 1999). Then, in the present study, we hypothesised that lower levels of EPA and DHA in serum phospholipids were associated with major depression and minor depression in lung cancer patients. However, the result of the present study did not support the hypothesis. This might have been caused by the relatively small rate of converting ALA to EPA and DHA, which was suggested in a previous study in healthy adults (Pawlosky *et al*, 2001). The converting rate might be so small that the reduction of ALA intake did not influence the reduction of the levels of EPA and DHA in serum phospholipids.

Previous studies reported that levels of EPA and DHA in serum or in the red blood cell membrane were lower in subjects with major depression than in healthy controls (Edwards *et al*, 1998; Peet *et al*, 1998; Maes *et al*, 1999). In the present study, there were no differences in the levels of DHA and EPA in serum phospholipids between subjects with major depression and subjects without depression. There are several possible explanations for the discrepancy in our findings. First, characteristics of depression may explain the difference in results of the present study from those of the previous studies. In the present study, all subjects had a clear stressful event, which was cancer diagnosis. Major depression in patients with cancer appears to be a reactive depression induced by the stressful events caused by and related to cancer (e.g., diagnosis, examination, therapy, and so on) (Chochinov, 2001; Moorey and Greer, 2002). To date, one study has reported no difference in the level of DHA in plasma phospholipids between reactive depression diagnosed by clinical interview and healthy controls (Fehily *et al*, 1981). Therefore, there is a possibility that the lack of any association between major depression and FAs in serum phospholipids in the present study was related to the unique characteristic of depression in cancer patients, such as the existence of clear stress and the patient's reaction to it. However, further research is needed before any definitive conclusion can be reached.

Second, the different proportions of males and females in subjects may be related to the different results. In the previous studies, the ratio of males to females was 1 : 1 or smaller. In the present study, about 70% of the subjects were males. A recent population-based cross-sectional study nested to the ATBC study showed no association between the intake of omega-3 FAs and depression in Finnish males (Hakkarainen *et al*, 2004). Besides, the other Finnish study reported a significant association between the frequency of fish intake and depression in females, but not in males (Tanskanen *et al*, 2001). These studies suggested the possibility that there may be no relation between omega-3 FAs and depression in males, which is consistent with our results. As subanalyses, the stratified analyses by gender showed no significant associations between FAs and depression in both genders. However, as the statistical power of the subanalysis was diminished due to reduced sample size particularly in females, further research is needed before a solid conclusion can be reached.

Third, the difference in dietary habits may contribute to the difference in results of the present study from those of the previous studies. In the present study, all subjects were Japanese, whose dietary habits are different from those in Western subjects, particularly in the elderly (Sugano and Hirahara, 2000), and who are reported to have relatively high intakes and high serum levels of omega-3 FAs (more than three times those of Caucasian Americans) (Iso *et al*, 1989). Hibbeln reported a higher consumption of seafood and a lower prevalence of major depression in Japan than in Western countries (Hibbeln, 1998). The small amount of EPA and DHA, which was hypothesised as one of the mechanisms of causing major depression in previous reports, may have had little contribution towards developing major depression in Japanese populations, who consume large quantities of seafood.

Contrary to our hypothesis, the level of DHA in serum phospholipids was significantly higher in subjects with minor depression. Few studies have investigated the association between minor depression and FAs. Maes *et al* (1996) performed a study to compare FAs of serum phospholipids among three groups: a group of 36 subjects with major depression, a group of 24 healthy controls, and a group of 14 subjects with minor depression including adjustment disorder with depressive mood and dysthymic disorder. Although the numbers of subjects with minor depression in the study might be too small to show any significant difference, the mean level of DHA in subjects with minor depression seemed to be about 10% above of that of subjects with major depression and healthy controls. The levels of DHA in serum phospholipids in minor depression may be higher than those in major depression and healthy controls. These findings suggested that minor depression, including adjustment disorder, dysthymic disorder, and the other subclinical form of depression, might have a biological pathology on FAs different from that of major depression.

The present study had the following limitations: (1) Subjects with severe depression might be excluded in this study, because subjects with poor physical activity, with cognitive impairment, with worse performance status (⩾2), and on current antidepressant medication were ineligible in this study. The inclusion of these subjects might have affected the result. (2) Owing to the lack of any data of FA compositions in the healthy controls, the influences of lung cancer on serum FAs were unclear. (3) Depression was defined by cutoff scores of the HADS-D, not by a structured psychiatric interview (such as the Structured Clinical Interview for DSM-IV, a widely recognised standard). The one point assessment of HADS-D on its own might not be enough to measure depression in the present study. (4) A previous study reported that serum FAs were reflected by dietary FA intake in the past 3 weeks (Zock *et al*, 1997). In the present study, the durations of all subjects between the time points of performing the HADS and blood sampling had a mean value of 3.6 days and standardised deviation of 5.0 days, and those of 95% of all subjects were under 15 days. In addition, the durations of all subjects were not different among the three compared groups. However, the influence of the durations cannot be totally excluded. (5) Although the subjects in the present study were matched by gender, age, clinical staging, and performance status, the present study was unable to exclude all of the confounding factors such as medications, smoking, alcohol consumption, and common physical complications.

In conclusion, the levels of DHA and EPA in serum phospholipids were not low in subjects with major and minor depressions in Japanese lung cancer patients. This result was not enough to encourage us to plan any further studies on interventional research with these FAs for depression in lung cancer patients. However, minor depression may be related to a high level of DHA in serum phospholipids. A further study with healthy controls and designed to exclude confounding factors is needed before any definitive conclusion can be reached.

## Figures and Tables

**Table 1:** Background of matching subjects(*n*=243)

	**Nondepression**	**Minor depression**	**Major depression**	***χ*^2^ or *F***	***P*-value**
HADS-D (score)	0–4	5–10	11–21		
Number	81	81	81		
Gender (male)	57 (70%)	57 (70%)	57 (70%)	0.00	1.00
Age (years old)	65.0±8.3	65.4±8.4	65.1±8.3	0.05^*^	0.96
Performance status (0/1)^a^	23/58	23/58	23/58	0.00	1.00
Clinical stage (IIIb–IV)^b^	47 (58%)	47 (58%)	47 (58%)	0.00	1.00
Educational level (>9 years)	56 (69%)	50 (62%)	52 (64%)	1.24	0.54
Alcohol (>45 g day^−1^)	12 (15%)	12 (15%)	14 (17%)	0.51	0.77
					
*Smoking*					
Current smoker	30 (37%)	27 (33%)	33 (41%)	1.32	0.86
Ex-smoker	31 (38%)	34 (42%)	32 (40%)		
Nonsmoker	20 (25%)	20 (25%)	16 (20%)		
					
Body mass index (kg m^−2^)	22.1±3.2	22.1±2.9	22.0±3.5	0.04^*^	0.96

Age and body mass index: mean±s.d. PS: number. Others: number and percentage. ^*^F-value.

a Defined by Eastern Cooperative Oncology Groups.

b Defined by TNM Classification, International Union Against Cancer.

**Table 2:** Percentage of omega-3 fatty acids in serum phospholipids in three groups

	**Depression**					**Compared groups**		
	**Non (*n*=81)**	**Minor (*n*=81)**	**Major (*n*=81)**			**Non *vs* Minor**	**Non *vs* Major**	**Minor *vs* Major**
**Fatty acids (FAs)**	**Mean±s.d.**	**Mean±s.d.**	**Mean±s.d.**	***F*-value**	***P*-value**	***P*-value^a^**	***P*-value^a^**	***P*-value^a^**
*Omega-3 FAs (%)*								
20:5n-3 (EPA)	2.96±1.53	3.24±1.45	2.92±1.33	1.16	0.31	—	—	—
22:5n-3	1.00±0.31	1.11±0.36	0.95±0.29	5.29	0.006	0.08	0.53	0.005
22:6n-3 (DHA)	7.40±1.54	7.90±1.40	7.25±1.52	4.15	0.017	0.09	0.79	0.017
Total omega-3	11.73±2.84	12.63±2.70	11.50±2.62	3.91	0.021	0.09	0.85	0.023

a *Post-hoc* analysis (Tukey HSD) was carried out only for fatty acids with significant difference. EPA: eicosapentaenoic acid; DHA: docosahexaenoic acid.

## References

[bib1] Akizuki N, Okamura H, Akechi T, Nakano T, Yoshikawa E, Nakanishi T, Uchitomi Y (2002) Clinical experience of the pharmacological treatment algorithm for major depression in advanced cancer patients: preliminary study. Int J Psychiatry Clin Pract 6: 83–89 24931933 10.1080/136515002753724072

[bib2] Bligh EG, Dyer WJ (1959) A rapid method of total lipid extraction and purification. Can J Biochem 37: 911–917 13671378 10.1139/o59-099

[bib3] Bruinsma KA, Taren DL (2000) Dieting, essential fatty acid intake, and depression. Nutr Rev 58: 98–108 10835899 10.1111/j.1753-4887.2000.tb07539.x

[bib4] Chochinov HM (2001) Depression in cancer patients. Lancet Oncol 2: 499–505 11905726 10.1016/S1470-2045(01)00456-9

[bib5] Derogatis LR, Morrow GR, Fetting J, Penman D, Piasetsky S, Schmale AM, Henrichs M, Carnicke Jr CL (1983) The prevalence of psychiatric disorders among cancer patients. JAMA 249: 751–757 6823028 10.1001/jama.249.6.751

[bib6] Edwards R, Peet M, Shay J, Horrobin D (1998) Omega-3 polyunsaturated fatty acid levels in the diet and in red blood cell membranes of depressed patients. J Affect Disord 48: 149–155 9543204 10.1016/s0165-0327(97)00166-3

[bib7] Fehily AMA, Bowey OAM, Ellis FR, Meade BW (1981) Plasma and erythrocyte membrane long chain polyunsaturated fatty acids in endogenous depression. Neurochem Int 3: 37–42 20487806 10.1016/0197-0186(81)90047-4

[bib8] Fisch M (2004) Treatment of Depression in Cancer. J Natl Cancer Inst Monogr 32: 105–111 10.1093/jncimonographs/lgh011 15263050

[bib9] Frasure-Smith N, Lesperance F, Julien P (2004) Major depression is associated with lower omega-3 fatty acid levels in patients with recent acute coronary syndromes. Biol Psychiatry 55: 891–896 15110732 10.1016/j.biopsych.2004.01.021

[bib10] Hakkarainen R, Partonen T, Haukka J, Virtamo J, Albanes D, Lonnqvist J (2004) Is low dietary intake of omega-3 fatty acids associated with depression? Am J Psychiatry 161: 567–569 14992986 10.1176/appi.ajp.161.3.567

[bib11] Hibbeln JR (1998) Fish consumption and major depression. Lancet 351: 1213 10.1016/S0140-6736(05)79168-6 9643729

[bib12] Innis SM (2000) The role of dietary n-6 and n-3 fatty acids in the developing brain. Dev Neurosci 22: 474–480 11111165 10.1159/000017478

[bib13] Iso H, Sato S, Folsom AR, Shimamoto T, Terao A, Munger RG, Kitamura A, Konishi M, Iida M, Komachi Y (1989) Serum fatty acids and fish intake in rural Japanese, urban Japanese, Japanese American and Caucasian American men. Int J Epidemiol 18: 374–381 2767851 10.1093/ije/18.2.374

[bib14] Kobayashi M, Sasaki S, Kawabata T, Hasegawa K, Akabane M, Tsugane S (2001) Single measurement of serum phospholipid fatty acid as a biomarker of specific fatty acid intake in middle-aged Japanese men. Eur J Clin Nutr 55: 643–650 11477462 10.1038/sj.ejcn.1601194

[bib15] Kugaya A, Akechi T, Okuyama T, Okamura H, Uchitomi Y (1998) Screening for psychological distress in Japanese cancer patients. Jpn J Clin Oncol 28: 333–338 9703862 10.1093/jjco/28.5.333

[bib16] Maes M, Christophe A, Delanghe J, Altamura C, Neels H, Meltzer HY (1999) Lowered omega3 polyunsaturated fatty acids in serum phospholipids and cholesteryl esters of depressed patients. Psychiatry Res 85: 275–291 10333380 10.1016/s0165-1781(99)00014-1

[bib17] Maes M, Smith R, Christophe A, Cosyns P, Desnyder R, Meltzer H (1996) Fatty acid composition in major depression: decreased omega 3 fractions in cholesteryl esters and increased C20: 4 omega 6/C20:5 omega 3 ratio in cholesteryl esters and phospholipids. J Affect Disord 38: 35–46 8735157 10.1016/0165-0327(95)00092-5

[bib18] McDaniel JS, Musselman DL, Porter MR, Reed DA, Nemeroff CB (1995) Depression in patients with cancer. Diagnosis, biology, and treatment. Arch Gen Psychiatry 52: 89–99 7848055 10.1001/archpsyc.1995.03950140007002

[bib19] Moorey S, Greer S (2002) Cognitive techniques 1: basic cognitive techniques. In Cognitive Behaviour Therapy for People with Cancer, pp 103–130. New York: Oxford University Press

[bib20] Nemets B, Stahl Z, Belmaker RH (2002) Addition of omega-3 fatty acid to maintenance medication treatment for recurrent unipolar depressive disorder. Am J Psychiatry 159: 477–479 11870016 10.1176/appi.ajp.159.3.477

[bib21] Ng KF, Innis SM (2003) Behavioral responses are altered in piglets with decreased frontal cortex docosahexaenoic acid. J Nutr 133: 3222–3227 14519814 10.1093/jn/133.10.3222

[bib22] Pawlosky RJ, Hibbeln JR, Novotny JA, Salem Jr N (2001) Physiological compartmental analysis of alpha-linolenic acid metabolism in adult humans. J Lipid Res 42: 1257–1265 11483627

[bib23] Peet M, Horrobin DF (2002) A dose-ranging study of the effects of ethyl-eicosapentaenoate in patients with ongoing depression despite apparently adequate treatment with standard drugs. Arch Gen Psychiatry 59: 913–919 12365878 10.1001/archpsyc.59.10.913

[bib24] Peet M, Murphy B, Shay J, Horrobin D (1998) Depletion of omega-3 fatty acid levels in red blood cell membranes of depressive patients. Biol Psychiatry 43: 315–319 9513745 10.1016/s0006-3223(97)00206-0

[bib25] Razavi D, Stiefel F (1994) Common psychiatric disorders in cancer patients. I. Adjustment disorders and depressive disorders. Support Care Cancer 2: 223–232 8087440 10.1007/BF00365726

[bib26] Su KP, Huang SY, Chiu CC, Shen WW (2003) Omega-3 fatty acids in major depressive disorder. A preliminary double-blind, placebo-controlled trial. Eur Neuropsychopharmacol 13: 267–271 12888186 10.1016/s0924-977x(03)00032-4

[bib27] Sugano M, Hirahara F (2000) Polyunsaturated fatty acids in the food chain in Japan. Am J Clin Nutr 71(Suppl): s189–s196 10.1093/ajcn/71.1.189S 10617970

[bib28] Suzuki S, Akechi T, Kobayashi M, Taniguchi K, Goto K, Sasaki S, Tsugane S, Nishiwaki Y, Miyaoka H, Uchitomi Y (2004) Daily omega-3 fatty acid and depression in Japanese patients with newly diagnosed lung cancer. Br J Cancer 90: 787–793 14970854 10.1038/sj.bjc.6601621 PMC2410186

[bib29] Tanskanen A, Hibbeln JR, Tuomilehto J, Uutela A, Haukkala A, Viinamaki H, Lehtonen J, Vartiainen E (2001) Fish consumption and depressive symptoms in the general population in Finland. Psychiatr Serv 52: 529–531 11274502 10.1176/appi.ps.52.4.529

[bib30] Tsubono Y, Takamori S, Kobayashi M, Takahashi T, Iwase Y, Iitoi Y, Akabane M, Yamaguchi M, Tsugane S (1996) A data-based approach for designing a semiquantitative food frequency questionnaire for a population-based prospective study in Japan. J Epidemiol 6: 45–53 8795957 10.2188/jea.6.45

[bib31] Zeleniuch-Jacquotte A, Chajes V, Van Kappel AL, Riboli E, Toniolo P (2000) Reliability of fatty acid composition in human serum phospholipids. Eur J Clin Nutr 54: 367–372 10822282 10.1038/sj.ejcn.1600964

[bib32] Zigmond AS, Snaith RP (1983) The hospital anxiety and depression scale. Acta Psychiatr Scand 67: 361–370 6880820 10.1111/j.1600-0447.1983.tb09716.x

[bib33] Zock PL, Mensink RP, Harryvan J, de Vries JH, Katan MB (1997) Fatty acids in serum cholesteryl esters as quantitative biomarkers of dietary intake in humans. Am J Epidemiol 145: 1114–1122 9199541 10.1093/oxfordjournals.aje.a009074

